# Prevalence of chronic pain in hemodialysis patients and its correlation with C-reactive protein: a cross-sectional study

**DOI:** 10.1038/s41598-023-32648-8

**Published:** 2023-03-31

**Authors:** Aya Mizher, Heba Hammoudi, Farah Hamed, Abrar Sholi, Adham AbuTaha, Mazen A. Abdalla, Mohammad M. Jaber, Mohannad Hassan, Amer A. Koni, Sa’ed H. Zyoud

**Affiliations:** 1 Department of Medicine, College of Medicine and Health Sciences, An-Najah National University, Nablus, 44839 Palestine; 2 Department of Biomedical Sciences, Faculty of Medicine and Health Sciences, An-Najah National University, Nablus, 44839 Palestine; 3 Department of Pathology, An-Najah National University Hospital, Nablus, 44839 Palestine; 4 Department of Orthopedic Surgery, An-Najah National University Hospital, Nablus, 44839 Palestine; 5 Department of Nephrology, An-Najah National University Hospital, Nablus, 44839 State of Palestine; 6 Division of Clinical Pharmacy, Department of Hematology and Oncology, An-Najah National University Hospital, Nablus, 44839 Palestine; 7 Department of Clinical and Community Pharmacy, College of Medicine and Health Sciences, An-Najah National University, Nablus, 44839 Palestine; 8 Poison Control and Drug Information Center (PCDIC), College of Medicine and Health Sciences, An-Najah National University, Nablus, 44839 Palestine; 9 Clinical Research Center, An-Najah National University Hospital, Nablus, 44839 Palestine

**Keywords:** Biomarkers, Kidney diseases

## Abstract

End-stage renal disease (ESRD) is a common chronic disease worldwide that requires hemodialysis. Patients may face chronic pain and poor quality of life. Therefore, a better understanding of these variables in hemodialysis patients is essential to provide a good intervention. We aim to determine how common chronic pain is in hemodialysis patients and its correlation with sociodemographics, C-reactive protein (CRP), calcium, phosphorus, albumin, and parathyroid hormone. A cross-sectional study of hemodialysis patients was conducted in Palestine. Data collection took place between November 2020 and May 2021. We used the brief pain inventory score to assess chronic pain, and lab tests detected CRP levels. Data were collected using a convenience sampling technique. There were two hundred sixty-one patients in the present study. The mean age of the patients was 51 years, with 63.6% being men. 47.1% of them reported having chronic pain. Gender (*p* = 0.011), social status (*p* = 0.003), educational status (*p* = 0.010), and number of chronic diseases (*p* = 0.004) indicated a significant relationship with the severity score of pain. Furthermore, sex (*p* = 0.011), social status (*p* = 0.003), and number of chronic diseases (*p* = 0.002) were significantly associated with the pain interference score. Additionally, Person’s test indicated significant correlations between CRP and pain severity (*p* < 0.001) and with pain interference (*p* < 0.001). Albumin was significantly and negatively correlated with pain severity (*p* = 0.001) and pain interference (*p* < 0.001). Multiple linear regression analysis revealed that patients who had a higher CRP level and many chronic diseases were more likely to have a higher pain severity score. However, pain severity was the only predictor for pain interference. Our results suggest that there is a significant correlation between the existence of chronic pain in hemodialysis patients and increased CRP levels. However, further investigations are needed with a larger number of patients in more than one dialysis unit to confirm this correlation and management of chronic pain in patients with HD.

End-stage renal disease (ESRD) or chronic kidney disease (CKD) is a term that refers to all stages of renal impairment, from damaged at risk to mild, moderate, and severe chronic kidney failure^1^. Patients with CKD receive integrated care, including kidney replacement therapy (KRT), which can be dialysis or kidney replacement, and non-KRT conservative care^2^. Almost 90% of patients receiving hemodialysis (HD) are considered the most common kind of KRT^3^. Hemodialysis is used primarily to treat acute and chronic renal failure that has not responded to conventional medical therapy^4^. Despite the great importance of hemodialysis in treating ESKD, hemodialysis has many side effects in these patients. Patients who receive chronic hemodialysis sessions regularly may develop a range of consequences, including intradialytic cardiovascular instability, malnutrition, and vascular access problems^4^. Furthermore, there is growing evidence of the correlation between hemodialysis and chronic pain among these patients^5^.

Pain is described as a physically or emotionally uncomfortable experience that can lead to impotence, low physical activity, anxiety, and interruptions in tasks and social relationships^6^. Pain in patients with HD is usually moderate to severe. Neuropathic, visceral, somatic, neuropathic, nociceptive, and complicated regional pain syndromes are all factors that contribute to pain^7^. A study in patients with HD showed that 55% had severe pain in the previous day, and three-quarters of them reported inadequate pain management^5^. Psychological difficulties, disturbed sleep, lower dialysis compliance, and a decreased quality of life can all be due to inadequately controlled pain in patients with HD^8–10^. In addition, chronic pain was reported to be strongly associated with all-cause mortality in CKD patients^11^.

C-reactive protein (CRP) is an indicator of the underlying inflammatory process in ESRD; it is also a precise objective measure of inflammatory activity, accurately reflecting the production of proinflammatory cytokines, including tumor necrosis factor-a (TNF-a) and interleukin (IL-6)^12^. Importantly, disturbed parameters such as calcium, vitamin, parathyroid hormone, and CRP were significantly related to chronic pain in patients with HD^13^. However, certain articles did not show a significant difference in the level of CRP between patients with/without pain^10,11^. These debates urge the need to explore more about this issue, as recommended by a recent study^10^.

Some studies on HD patients in Palestine were conducted and published regarding the use of complementary and alternative medicaine and the quality of life of this population^14,15^. Nevertheless, no single study has emphasized the prevalence of chronic pain in hemodialysis patients and its correlation with reactive protein. Therefore, the primary aim of the current study was to clinically describe HD patients, detect the prevalence of chronic pain among them, and determine the variables influencing the prevalence of chronic pain in hemodialysis patients. In addition, our secondary aim was to detect whether there is a correlation between laboratory tests (CRP, albumin, phosphorus, calcium, and parathyroid hormone (PTH)) and chronic pain.

This research will help to conduct further research on hemodialysis patients with chronic pain and to provide recommendations that can help hemodialysis patients control chronic pain, which leads to a higher quality of life. Furthermore, this study will provide new data on the value of chronic pain among hemodialysis patients and its relationship with CRP levels, which will help better understand the factors that affect chronic pain in patients with HD.

## Methods

### Study design

A cross-sectional design was adopted to achieve the objectives of the study. Data collection took place between November 2020 and May 2021.

### Study setting

The research was carried out at An-Najah National University Hospital in Nablus, Palestine. The hospital has a specialized dialysis unit that treats more than 300 patients.

### Sampling procedure, study population, and sample size calculation

According to the report of the Palestinian Ministry of Health for 2019, An-Najah National University Hospital had 350 registered patients who were receiving dialysis on a regular basis^16^. The sample size for this study was calculated using the Raosoft sample size calculator, with a 350 HD population, a 50% expected response, a 95% confidence interval, and a 5% error margin. The minimum number of patients required in this study was 184 HD patients. We recruited and interviewed 280 patients using convenience sampling.

### Inclusion and exclusion criteria

All hemodialysis outpatients who had been on dialysis for at least eight weeks, were aged over 18 years, and had completed the Institutional Review Board's consent form were chosen to participate in our study. Patients who were mentally or physically unable to communicate with the interviewer were excluded.

### Data collection tool

The data collection tool used contained three parts. Part 1 consisted of sociodemographic factors, including sex, age, occupation, residency, smoking status, education level, monthly income, and marital status. Part 2 contained the clinical status of the patients, including the number of hemodialysis sessions per week, the number of years of dialysis, body mass index (BMI), the number of chronic diseases, the number of medications used, and CRP value. The CRP levels from either serum or plasma fluid were analysed using the particle enhanced immunoturbidimetric assay method. Weight before and after dialysis is probable, thus affecting BMI. Therefore, the dry weight of the patients was measured.

In the third part, we included an instrument for measuring pain. It was the Arabic version of theBrief Pain Inventory (BPI), which is a well-known tool for measuring pain in terms of its intensity (sensory dimension) and how much it interferes with the patient’s life (reactive dimension)^17^. We were granted authorization to use the validated Arabic version of the short form BPI that the MD Anderson Cancer Center offers^17–20^. The BPI scale uses basic numeric rating scales (NRS) ranging between zero and ten. It defines pain as follows: worst pain: 1 – 4 indicates mild pain; worst pain: 5 – 6 indicates moderate pain; and worst pain: 7–10 indicates severe pain. The BPI asks patients to rate their pain when they take the questionnaire. This is because pain levels are susceptible to change throughout the day. In addition to the former, the respondent must describe the pain level they had felt in the previous week, ranging between worst, least, and average. The BPI (short form) is a 9-point questionnaire focused on pain and related issues.

### Statistical analysis

The Statistical Package for Social Sciences program version 15 (SPSS) was used to enter and then analyse the data. Frequencies (percentages) were used to represent categorical variables. The medians (lower–upper quartiles) were used to represent variables that were not normally distributed. The normality of the variables was determined using the Kolmogorov‒Smirnov test. The Mann‒Whitney and Kruskal‒Wallis tests were used to test for differences in medians. Pearson's correlation was applied to test the relationship between CRP and pain scores. Furthermore, we performed multiple linear regression analyses for all significant variables. A P value < 0.05 was selected as the significance number.

### Ethical approval

The Institutional Review Board (IRB) *of An-Najah National University* and local health authorities approved all aspects of the study procedure on 24th November 2020 (Ref: Med. Nov. 2020/27). Permission from An-Najah National University Hospital was given to facilitate data collection. Additionally, all participants who agreed to participate in this study provided consent. We confirm that the gathered information was only used for research, and the provided data will be confidential and only be used for this research. An informant constant was given to all participants that confirmed the privacy of the data and that all the data would be secret and used only for research purposes. Because we did not collect any identifying information during the interview and our study did not pose a major risk to participants, the *IRB of An-Najah National University* waived the requirement for written informed consent. Therefore, the *IRB of An-Najah National University* approved only verbal informed consent from study participants. We confirm that all experiments and methods were performed in accordance with relevant guidelines and regulations.

## Results

### Demographic and clinical characteristics

In total, 280 patients were asked to participate in the survey, and 261 accepted and were included in the definitive analysis (response rate, 93.2%). The average age of the patients was 51 years, with 74.3% of the participants over 50 years old. Males 63.6%, married 77%, and city dwellers 46.4% made up the majority of participants. The subjects' unemployment rate was high, 84.7%, and 61.7% of them had an income of less than 2000 New Israeli Shekels (1 NIS = 0.30 US dollars) per month. Only 10.7% of participants were without an educational degree. Regarding the variables related to dialysis, 68.2% received dialysis for less than four years, and 92.7% underwent three sessions per week. Most of them (91.2%) spent more than four hours in each session. Only 8.8% of the participants had previously undergone a kidney transplant. Almost half of the 47.9% had three or more chronic comorbidities, but most of them (82%) were on four or more chronically used medications. Finally, 24.5% are current smokers. The mean ± SD for CRP, albumin, calcium, phosphorus and parathyroid hormone was 14.6 ± 23.4, 3.6 ± 0.5, 8.7 ± 0.9, 4.7 ± 1.5, and 451.6 ± 453.7, respectively. The personal, social, and clinical information of the patients is attached in Table 1 in detail.

**Table 1:** Sociodemographic and clinical characteristics of the study sample.

Variable		Frequency (%)
		N = 261
Gender	Male	166 (63.6)
	Female	95 (36.4)
Age category (Years)	18–29	16 (6.1)
	30–49	51 (19.5)
	50–69	129 (49.4)
	≥ 70	65 (24.9)
Marital status	Single, divorced, widow	60 (23)
	Married	201 (77)
BMI	Underweight	15 (5.7)
	Normal weight	86 (33)
	Overweight	85 (32.6)
	Obese & morbid obese	75 (28.7)
Income	< 2000 NIS	161 (61.7)
	2000–5000 NIS	89 (34.1)
	5000–10,000 NIS	11 (4.2)
Residency	City	121 (46.4)
	Village	113 (43.3)
	Camp	27 (10.3)
Educational status	Uneducated	28 (10.7)
	Primary	120 (46)
	High school	61 (23.4)
	University	52 (19.9)
Occupation	Employed	40 (15.4)
	Unemployed	221 (84.7)
Dialysis Years	≤ 4 years	178 (68.2)
	> 4 years	83 (31.8)
Frequency of dialysis per week	≤ 2/week	15 (5.7)
	3/week	242 (92.7)
	> 3/week	4 (1.5)
Hours of dialysis per session	< 4 h	238 (91.2)
	≥ 4 h	21 (8.4)
History of kidney transplant	Yes	23 (8.8)
	NO	238 (91.2)
Number of chronic diseases	< 3 diseases	136 (52.1)
	≥ 3 diseases	125 (47.9)
Number of medications	< 4 drugs	47 (18)
	≥ drugs	214 (82)
Smoker	Yes	64 (24.5)
	No/previously a smoker	197 (75.5)
CRP	< 3	56 (21.5)
	≤ 3	205 (78.5)

*BMI,* body mass index; *NIS,* new Israeli shekel (1NIS = 0.30 US dollars).

### Presence, site, and management of pain

Approximately (47.1%) of the participants expressed chronic pain. Most reported pain in the lower back region (14.9%). However, approximately 13% had foot pain, 10.3% complained of upper arm pain, 11.5% had knee pain, 6.5% had thigh pain, 6.5% had chest pain, 6.1% had upper back pain, 5.4% had abdominal pain, and 5% had neck pain. 69.9% of the patients mentioned above reported using pain relief medications, most commonly paracetamol. In addition, approximately 12% of HD patients had pain at two sites, and 11.5% had three or more pain sites.

### Pain severity score

The associations of the variables with the pain severity score are shown in Table 2. CRP levels (*p* < 0.001), sex (*p* = 0.001), social status (*p* = 0.016), educational status (*p* = 0.013), and number of chronic diseases (*p* = 0.004) were significantly associated with pain severity score. Uneducated patients had the highest median severity score, 14.5 (0.75–20), among other groups of variable educational status. Patients with three chronic diseases had a higher median pain severity score, 6 (0–18.75), than those with fewer than three comorbidities. No significant associations were found between pain severity and dialysis-related factors such as years of dialysis, the duration of dialysis per session, and the number of sessions per week.

**Table 2:** Association between sociodemographic variables and pain severity (N = 261).

Variable		Frequency (%) N = 261	Mean Rank	Median (Q1-Q3)	p value*
Gender	Male	166 (63.6)	123.82	0 (0–13)	**0.04**
	Female	95 (36.4)	142.11	5 (0- 18)	
Age category (Years)	18–29	16 (6.1)	128.33	0 (0–17)	0.198
	30–49	51 (19.5)	118.8	0 (0–8)	
	50–69	129 (49.4)	127.88	0 (0–14)	
	≥ 70	65 (24.9)	145.38	9 (0–20.5)	
Marital status	Single, divorced, widow	60 (23)	149.55	9 (0–20)	**0.016**
	Married	201 (77)	124.91	0 (0–13)	
BMI	Underweight	15 (5.7)	159.5	12 (0–25)	0.21
	Normal weight	86 (33)	130.6	0 (0–13)	
	Overweight	85 (32.6)	121.16	0 (0–15)	
	Obese and morbid obese	75 (28.7)	135.17	0 (0–18)	
Income	< 2000 NIS	161 (61.7)	134.88	0 (0–18)	0.252
	2000–5000 NIS	89 (34.1)	125.91	0 (0–13.75)	
	5000–10,000 NIS	11 (4.2)	103.09	0 (0–6)	
Residency	City	121 (46.4)	123.98	0 (0–13)	0.309
	Village	113 (43.3)	134.36	1 (0–17)	
	Camp	27 (10.3)	143.35	3 (0–21)	
Educational status	Uneducated	28 (10.7)	168.54	14.5 (0.75–20)	**0.013**
	Primary	120 (46)	127.41	0 (0–17)	
	High school	61 (23.4)	131.0	0 (0–15)	
	University	52 (19.9)	116.78	0 (0–11.5)	
Occupation	Employed	40 (15.4)	133.23	0 (0–17)	0.136
	Unemployed	221 (84.7)	115.49	0 (0–12.75)	
Dialysis Years	≤ 4 years	178 (68.2)	126.92	0 (0–15)	0.223
	> 4 years	83 (31.8)	138.14	0 (0–20)	
Dialysis Sessions Per Week	≤ 2/week	15 (5.7)	162.23	14 (0–23)	0.182
	3/week	242 (92.7)	128.7	0 (0–15.5)	
	> 3/week	4 (1.5)	120.25	4.5 (0–11.25)	
Dialysis Hours/Session	< 4 h	238 (91.2)	130.18	0 (0–17)	0.893
	≥ 4 h	21 (8.4)	128.11	0 (0–17.75)	
History of kidney transplant	Yes	23 (8.8)	128.93	0 (0–17)	0.241
	NO	238 (91.2)	146.65	8 (0–17)	
Number of chronic diseases	< 3 diseases	136 (52.1)	118.71	0 (0–12)	**0.004**
	≥ 3 diseases	125 (47.9)	143.43	6 (0–18.75)	
Number of medications	< 4 drugs	47 (18)	124.09	0 (0–15)	0.483
	≥ 4drugs	214 (82)	131.92	0 (0–17)	
Smoker	Yes	64 (24.5)	123.61	0 (0–12)	0.359
	No/previously a smoker	197 (75.5)	132.75	0 (0–17)	
CRP	< 3	56 (21.5)	101.46	0 (0–4)	** < 0.001**
	≤ 3	205 (78.5)	138.47	5 (0–18)	

*BMI* body mass index; *CRP* C-reactive protein.

*The bold p value indicates that < 0.05, which considers significantly.

### Pain interference score

The associations of variables with the pain interference score are shown in Table 3. The level of CRP (*p* < 0.001), sex (*p* = 0.011), social status (*p* = 0.003), and number of chronic diseases (*p* = 0.002) were all significantly associated with the pain interference score. The pain interference score was 15 (0–46) for females compared to 0 (0–27.25) for males. The score was higher, 26 (0–45.75), for single social status than for married status, 0 (0–28). No significant associations were found between the pain interference score and the years of dialysis, the number of sessions per week, or the duration of dialysis per session.

**Table 3:** Pain interference score by subgroups based on demographic and clinical characteristics.

Variable		Frequency (%) N = 261	Mean Rank	Median (Q1-Q3)	p value*
Gender	Male	166 (63.6)	122.85	0 (0–27.25)	**0.011**
	Female	95 (36.4)	145.25	15 (0–46)	
Age category (Years)	18–29	16 (6.1)	133.59	0 (0–40.75)	0.282
	30–49	51 (19.5)	118.23	0 (0–25)	
	50–69	129 (49.4)	129.66	0 (0–35)	
	≥ 70	65 (24.9)	143.05	10 (0–41.5)	
Marital status	Single, divorced, widow	60 (23)	154.02	26 (0–45.75)	**0.003**
	Married	201 (77)	124.13	0 (0–28)	
BMI	Underweight	15 (5.7)	164.13	28 (0–59)	0.150
	Normal weight	86 (33)	131.77	0 (0–34)	
	Overweight	85 (32.6)	121.41	0 (0–27.5)	
	Obese & morbid obese	75 (28.7)	134.36	0 (0–38)	
Income	< 2000 NIS	161 (61.7)	135.93	0 (0–41.5)	0.285
	2000–5000 NIS	89 (34.1)	124.85	0 (0–26)	
	5000–10,000 NIS	11 (4.2)	108.55	0 (0–18)	
Residency	City	121 (46.4)	125.59	0 (0–30.5)	0.453
	Village	113 (43.3)	134.45	0 (0–39.5)	
	Camp	27 (10.3)	140.83	16 (0–43.0)	
Educational status	Uneducated	28 (10.7)	177.34	38 (4.5–38)	**0.001**
	Primary	120 (46)	127.6	0 (0–33.5)	
	High school	61 (23.4)	132.22	0 (0–32.5)	
	University	52 (19.9)	112.85	0 (0–18)	
Occupation	Employed	40 (15.4)	134.4	0 (0–37.5)	0.061
	Unemployed	221 (84.7)	112.2	0 (0–25.5)	
Dialysis Years	≤ 4 years	178 (68.2)	128.25	0 (0–34)	0.345
	> 4 years	83 (31.8)	136.89	0 (0–39)	
Frequency of dialysis per week	≤ 2/week	15 (5.7)	160.53	27 (0–49)	0.202
	3/week	242 (92.7)	128.61	0 (0–34)	
	> 3/week	4 (1.5)	146.88	19 (0–51)	
Hours of dialysis per session	< 4 h	238 (91.2)	130.96	0 (0–36.25)	0.720
	≥ 4 h	21 (8.4)	125.48	0 (0–34)	
History of kidney transplant	Yes	23 (8.8)	129.58	0 (0–36)	0.283
	No	238 (91.2)	145.72	20 (0–34)	
Number of chronic diseases	< 3 diseases	136 (52.1)	118.44	0 (0–24)	**0.002**
	≥ 3 diseases	125 (47.9)	144.67	0 (0–37)	
Number of medications	< 4 drugs	47 (18)	118.85	0 (0–24)	0.182
	≥ 4drugs	214 (82)	133.67	0 (0–37)	
Smoker	Yes	64 (24.5)	123.95	0 (0–24.75)	0.345
	No/previously a smoker	197 (75.5)	133.29	0 (0–38)	
CRP	< 3	56 (21.5)	101.36	0 (0–3)	** < 0.001**
	≤ 3	205 (78.5)	138.5	4.5 (0–40)	

*BMI* body mass index; *CRP* C-reactive protein.

*The bold *p* value indicates that < 0.05, which considers significantly.

### Correlations

The Pearson correlation coefficient values for the pain severity score with CRP and the pain interference score were 0.878 and 0.297, respectively, and 0.294 for CRP and the pain severity score (Table 4). Furthermore, the coefficient was -0.253 for pain interference and albumin and -0.200 for pain severity and albumin. These correlations were statistically significant for all studied variables: pain with pain interference (*p* < 0.001), CRP (*p* < 0.001), and albumin (*p* = 0.001) and pain interference with CRP (*p* < 0.001) and albumin (*p* < 0.001). However, other laboratory parameters (calcium, phosphorus, and parathyroid hormone) were not significantly correlated with either pain score (Table 4).

**Table 4:** Correlation between pain severity and pain interference with laboratory parameters (albumin, calcium, phosphorus, PTH, and CRP).

	Pain severity score	Albumin	Calcium	Phosphorus	PTH	CRP
*Pain interference*						
Pearson Correlation	0.878	−0.253	−0.043	0.020	0.077	0.297
P value*	** < 0.001**	** < 0.001**	0.495	0.751	0.217	** < 0.001**
*Pain severity score*						
Pearson Correlation	1	−0.200	−0.033	−0.039	0.083	0.294
P value*		**0.001**	0.598	0.527	0.182	** < 0.001**

*CRP* C-reactive protein; *PTH* parathyroid hormone.

*The bold p value indicates that < 0.05, which considers significantly.

### Multiple linear regression analysis

Our findings revealed that patients who have a higher CRP level and many chronic diseases are more likely to have a higher pain severity score. However, pain severity was the only predictor for pain interference (Tables 5 and 6).

**Table 5:** Multivariate linear regression analysis of the pain severity score.

Model		Unstandardized coefficients		Standardized coefficients	t	Sig.*	95.0% Confidence interval for B		95.0% Confidence interval for B
		B	SE	Beta			Lower Bound	Lower Bound	VIF
1	(Constant)	10.257	6.989		1.468	0.143	−3.506	24.021	
	Gender	2.287	1.460	.106	1.566	0.119	−.589	5.163	1.348
	Marital status	−.847	1.614	−.034	−.525	0.600	−4.027	2.332	1.246
	Education	−.415	.701	−.037	−.592	0.554	−1.794	.965	1.167
	Number of chronic diseases	2.632	1.244	.126	2.117	**0.035**	.183	5.081	1.052
	Albumin	−2.312	1.375	−.103	−1.681	0.094	−5.020	.397	1.116
	CRP	.116	.028	.262	4.233	** < 0.001**	.062	.171	1.128

a. Dependent Variable: pain severity.

*The bold p value indicates that < 0.05, which considers significantly.

**Table 6:** Multivariate linear regression analysis of the pain interference score.

Model		Unstandardized coefficients		Standardized coefficients	t	Sig.*	95.0% Confidence interval for B		95.0% Confidence interval for B
		B	SE	Beta			Lower Bound	Lower Bound	VIF
1	(Constant)	16.779	7.337		2.287	0.023	2.329	31.229	
	Gender	1.154	1.534	.025	.753	0.452	−1.867	4.176	1.361
	Marital status	−2.745	1.689	−.053	−1.625	0.105	−6.070	.581	1.248
	Education	−1.199	.733	−.051	−1.637	0.103	−2.643	.244	1.169
	Number of chronic diseases	1.652	1.311	.038	1.259	0.209	−.931	4.235	1.070
	Albumin	−2.838	1.446	−.060	−1.963	0.051	−5.685	.009	1.128
	CRP	.020	.030	.021	.662	0.508	−.039	.078	1.208
	Pain severity	1.753	.066	.836	26.677	** < 0.001**	1.624	1.883	1.167

a. Dependent Variable: pain interference.

*The bold *p* value indicates that < 0.05, which considers significantly.

## Discussion

To our knowledge, until now, this is the first study of its kind in Palestine that examines the association between laboratory data and chronic pain in HD patients. CRP is a sensitive and independent marker of anemia, malnutrition, and amyloidosis, all of which can increase hemodialysis patients' perception of pain^21^. Furthermore, the mortality risk in patients undergoing HD increased significantly with high CRP levels^22^. This study is likely to help healthcare practitioners better understand the variables correlated with the prevalence of chronic pain in hemodialysis patients.

Our current study used the brief pain inventory assessment tool to examine pain symptoms among patients undergoing HD in Palestine. The Brief Pain Inventory is a tool to assess pain in both clinical and research settings. In addition, we evaluated the relationship between chronic pain, CRP levels, and other laboratory parameters in HD patients.

According to our findings, 47% of our patients had abnormal chronic pain that interfered with their lives in our study. A lower percentage, 38%, was found among patients with HD^23^. This problem's prevalence was 52% in Egypt, which is similar to our finding^13^. Another analysis reported a prevalence of 74.4%, with the majority having neuropathic pain^10^. Furthermore, 89.23% in another publication reported severe pain, and 21.54% complained^24^. In our study, lower back pain was the most prevalent pain, while pain in the lower and upper trucks and limbs was the most frequently reported pain in a previous study^24^. However, headache was documented in 32% of HD patients^25^. Importantly, pain in HD patients was found to interfere with normal daily activities, work, social relationships, walking, and mood^24–26^. It has also been reported that pain in peritoneal dialysis patients is a determinant of depression, quality of life, and sleep quality^27^. These results showed that the prevalence of pain varies between different studies and the site of pain; therefore, this symptom should not be neglected and should be appropriately managed.

Gender, social status, education status, and the number of chronic diseases in the patient significantly impacted the severity score. Comorbidity and gender were previously documented to significantly impact chronic pain^28^. However, dialysis-related factors were not significantly associated with the severity of pain, which is in disagreement with a study that found that dialysis time was substantially related to pain^28^.

In our study, we measured the levels of CRP in HD patients and considered ≥ 3 as a high CRP result; 78.5% of our patients had high CRP levels. A previous study showed that using high-throughput hemodialysis had a significant role in decreasing CRP levels^29^. In addition, a study in Japan showed that 24.39% of HD patients had high CRP levels, which is significantly less than our study^30^. An important finding is that the correlations between pain severity and pain interference with CRP were 0.294 and 0.297, respectively, which are considered weak correlations. Furthermore, the CRP level was a determinant of pain severity. A previous study concluded that CRP can be examined as a potential biomarker for chronic pain^31^. However, a review study revealed that high CRP levels were in individuals with acute low back^32^, and another study found an association between CRP and acute pain but not with chronic pain^33^. In hemodialysis patients, high CRP levels are strongly correlated with chronic pain^13^. However, in another study, CRP levels were found to be similar between two groups of patients with CKD who were either with or without chronic musculoskeletal pain or without it^11^. Furthermore, a previous study showed a significant association between low calcium levels and high parathyroid value with chronic pain^13,27^. In contrast, we did not find a significant correlation between these variables. Additionally, the albumin level was found to be substantially associated with chronic pain^13^, similar to our results that showed that the albumin level was negatively and significantly correlated with pain. Further research is needed on the correlations between laboratory data and chronic pain in HD patients.

There were no significant differences in age, BMI score, smoking, or pain severity in HD patients. On the other hand, our patients had high statistical significance between gender, marital status, education status, occupation, comorbidities, and pain severity. In addition, a previous study conducted in more than one center in West Bank, Palestine, addressing persistent pain in HD patients showed that gender, BMI, and education status are more associated with the severity of pain^34^.

### Strengths and limitations

This study has many strengths, including that it is the first to examine the correlation between chronic pain and CRP levels in HD patients in Palestine. Furthermore, face-to-face interviews were used to collect data from participants, which may have increased the reliability of data collection. In addition, the laboratory results were taken directly from the hospital electronic system, and the data were entered into SPSS by two researchers each time to decrease the susceptibility to bias. However, because this is a cross-sectional study, it has some limitations. This means that in this study, we have taken a snapshot of the current situation, which may not accurately reflect long-term changes. Furthermore, other factors that could cause high CRP levels, e.g., acute infection, acute and chronic inflammation, postsurgery, chronic diseases (i.e., diabetes, malignant tumors, cardiovascular diseases, and arthritis) were not considered when the CRP level was obtained, which might affect the interpretation of the current findings.

## Conclusions

Our study shows that 78.5% of patients with HD had high CRP results, and 47% had abnormal chronic pain that interfered with their lives. This work suggests a weak and significant association between chronic pain in hemodialysis patients and higher levels of CRP. However, more studies are needed with a larger number of patients in more than one dialysis unit to confirm this correlation and improve the diagnosis and management of chronic pain in patients with HD. Furthermore, it is crucial to reveal the HD patients’ other comorbid conditions and carry out a subanalysis on these populations separately.

## Data Availability

Due to privacy, the data sets used and/or analysed during the current study are available from the corresponding author on reasonable request. This manuscript forms part of a Doctor of Medicine graduation project submitted to An-Najah National University. The abstract was published as part of self-archiving in institutional repositories (university repository: https://repository.najah.edu/handle/20.500.11888/16058).

## Abbreviations

ESRD: End-stage renal disease
CRP: C-reactive protein
CKD: Chronic kidney disease
KRT: Kidney replacement therapy
HD: Hemodialysis
IL-6: Interleukin
TNF-a: Tumor necrosis factor-a
BMI: Body mass index
NRS: Numeric rating scales
BPI: Brief Pain Inventory
IRB: Institutional Review Board
SPSS: Statistical Package for Social Sciences
